# Organizational Climate as a Key to Positive Mental Health and Academic Engagement in University Students: A Structural Equation Modeling Approach

**DOI:** 10.3390/ejihpe15020017

**Published:** 2025-02-02

**Authors:** Roger Pedro Norabuena-Figueroa, Hugo Marino Rodríguez-Orellana, Emerson Damián Norabuena-Figueroa, Angel Deroncele-Acosta

**Affiliations:** 1 Departamento Académico de Estadística, Facultad de Ciencias Matemáticas, Universidad Nacional Mayor de San Marcos, Lima 150101, Peru; rnorabuenaf@unmsm.edu.pe (R.P.N.-F.); hrodriguezo@unmsm.edu.pe (H.M.R.-O.); enorabuenaf@unmsm.edu.pe (E.D.N.-F.); 2 Doctorado en Educación, Escuela de Postgrado, Universidad San Ignacio de Loyola, Lima 15024, Peru

**Keywords:** education, health, psychology, students, university

## Abstract

This study aimed to examine the relationships between mental health, organizational climate, and engagement through a structural equation model, for which a quantitative methodology was developed. A total of 1971 students from a public university in Lima (between 16 and 56 years of age, with a mean age of 21.09 years and standard deviation of 3.2) participated in the study. Three instruments with high internal consistency were used: the Positive Mental Health Scale, the Utrecht Work Engagement Scale, and the FOCUS Organizational Climate Questionnaire. The results show that organizational climate is positively related to mental health and engagement, with life satisfaction being the most significant dimension in mental health (0.768), the vigor the strongest in engagement (0.814), and the innovation climate stood out in organizational climate (0.819). At the same time, mental health directly impacts engagement. The structural model revealed that organizational climate directly influences mental health (0.64) and engagement (0.49), while mental health has a direct impact on engagement (0.43). In addition, this structural model presents an adequate fit. The findings highlight the need to design interventions prioritizing students’ psychosocial well-being and managing a positive organizational climate as a premise. Implications of the study are discussed.

## 1. Introduction

The scientific literature reflects a negative association between positive mental health and academic stress (A. Khan et al., 2023). This implies that as students’ positive mental health increases, academic stress tends to decrease, and vice versa. In other words, students who enjoy greater emotional, psychological, and social well-being (i.e., good mental health) tend to experience less stress related to their studies. This type of relationship suggests that promoting positive mental health may be an effective strategy for reducing academic stress in students.

Several studies consistently show a negative relationship between stress and positive mental health in university students, highlighting that psychosocial well-being, when high, acts as a protective factor against academic stress and reduces suicidal ideation. In multiple international contexts, positive mental health moderates the impact of stress and improves students’ ability to cope with academic challenges, underscoring the need for wellness-strengthening interventions to mitigate the effects of chronic stress (Albaqawi et al., 2022; A. Khan et al., 2023; S. Khan & Shamama-Tus-sabah, 2020; Teismann et al., 2023; Zhou et al., 2022).

This is urgent, especially in a context where one in five college students is reported to struggle with suicidal ideation (Klonoff-Cohen et al., 2024), in a scenario where depression reappears again and again in students at this stage (Kim et al., 2021), and where chronic stress remains a reality for university students in many parts of the world (Latin America, Asia, Europe), on a sustained basis over time (Guangorena-Gómez et al., 2022; Gulewitsch et al., 2013; Pozos-Radillo et al., 2014; Zhang et al., 2020).

Along these same lines, a study found that positive mental health can help protect people from the effects of stress (Page et al., 2014), while another study argues that university health stakeholders should consider the buffering effects of protective factors on mental health (Peoples et al., 2022).

In this regard, positive psychology has emphasized positive protective factors (M. E. Seligman & Csikszentmihalyi, 2000; M. E. P. Seligman et al., 2009); among these are positive mental health (Bjørnsen et al., 2019; Halama & Dědová, 2007; Keyes, 2013; Lukat et al., 2016), engagement (Kotera & Ting, 2021; Ouweneel et al., 2013; Sweetman & Luthans, 2010), and organizational climate (Singha, 2024). However, despite the relevance of each of these variables, their interrelation has been scarcely explored, especially at the university level.

Positive mental health is associated with happiness, enjoyment, life satisfaction, self-confidence, effective autonomy, integral well-being, resilience, joy, and serenity (Boufellous et al., 2023; Lukat et al., 2016). Positive mental health is a useful adjunct in the surveillance and prediction of suicide risk and academic underachievement (Keyes et al., 2012). In this sense, promoting positive mental health not only has an impact on the individual level but also contributes to collective well-being by achieving healthy and resilient organizations (Salanova et al., 2012).

Positive mental health among university students has gained significant attention in recent studies globally, emerging as a crucial factor for their well-being and academic performance. Various studies have highlighted its importance in different international contexts, such as in South Africa (Vagiri et al., 2024), Italy (Mangialavori et al., 2024), India (Divecha & Acharya, 2024), Portugal (Valentim et al., 2023), China (Brailovskaia et al., 2022), Turkey (Çeçen & Vatandaşlar, 2021), Mexico (Fouilloux et al., 2021), Brazil (Sousa et al., 2021), United States (McDermott et al., 2021), Spain (Sequeira et al., 2020), Korea (Shin & Lim, 2019), and Hong Kong (Arat & Wong, 2019).

The synthesis of these studies shows that positive mental health (PMH) in university students is understood as an essential factor for their general well-being and academic success. The studies reviewed highlight several central themes: First, PMH includes psychological, emotional, and social dimensions that can be influenced by sociodemographic variables, lifestyles, and cultural factors. Healthy habits such as adequate sleep, physical activity, and healthy eating, along with skills such as resilience and self-care, are key determinants of high PMH.

In this context, the need for strategies that encourage flow and leisure activities is evident, especially to counteract risks such as stress, anxiety, and depression. At the same time, contextual factors such as occupation, religious affiliation, and academic characteristics have a significant impact on PMH. Students in medical and nursing disciplines, for example, face unique challenges due to academic demands and exposure to ongoing stress. However, promoting individual resources, such as self-compassion and social connectedness, can mitigate these risks. In sum, the studies underscore the importance of personalized and culturally sensitive approaches to supporting college students’ mental health and resilience, which calls for reflection on the focus of climate and culture in organizations.

An interesting study identified protective mechanisms associated with positive mental health (PMH) in college students, including spirituality, social support, self-esteem, and racial identity. These mechanisms are considered essential to promote the mental flourishing of Black students and enhance their overall well-being within the college context (Mushonga & Henneberger, 2020).

However, a study analyzing burnout and academic engagement, considered theoretical opposites, in university students from Spain, Portugal, and the Netherlands, shows that there is a negative correlation between burnout and engagement, confirming their inverse relationship. Moreover, regardless of the country, efficacy and vigor showed a positive relationship with academic performance. This underlines the need to implement interventions that reduce academic stress and promote practices that increase energy, dedication, and absorption in educational activities (Schaufeli et al., 2002). However, this is not a simple task and studies highlight the need to develop a broader understanding of engagement in support of improving the quality of the student experience (Krause & Coates, 2008).

Academic engagement is a construct that reflects the level of involvement and commitment of students to their educational process. To evaluate this research, this construct is understood from the interrelation of the processes of vigor, dedication, and absorption (Schaufeli et al., 2006). For this study, vigor refers to energy and vitality in academic work, dedication indicates the degree of involvement and enthusiasm for academic tasks, and absorption describes the state of immersion and concentration in learning.

Studies have addressed the impact of engagement on mental health (Grégoire et al., 2018; Kotera & Ting, 2021). It has been found that especially in virtual environments the climate of online learning can hurt both the engagement and mental health of students (Nicholson et al., 2023), while it has also been shown that the school environment can positively affect the mental health of college students through the mediating role of engagement in learning (Huang et al., 2024).

The above, recent studies highlight the significant impact of technology on mental health, showing how excessive use of digital technologies and social networks can trigger burnout and depressive symptoms, affecting both academic engagement and the psychological well-being of students (Liu & Ma, 2020; Salmela-Aro et al., 2017). These findings underscore the need to manage the use of these tools in educational settings and foster an organizational climate that limits their negative impact. The link between technological overexposure and mental health problems invites us to re-consider how technology is integrated into the daily lives of students and work dynamics in academic institutions, particularly in a context where digital technologies play a central and transversal role in daily dynamics.

This connotes that it is imperative to broaden the understanding of this relationship from a positive mental health perspective, assessing how technologies can not only be risk mitigators but also promoters of well-being. This would involve exploring the design of technological interventions that strengthen skills such as emotional self-regulation, resilience, and authentic social connection. Furthermore, it is crucial to investigate how individual factors, such as personality or coping styles, and specific sociocultural contexts, mediate the impact of technology, which implies today more than ever to reflect on the impact of artificial intelligence on positive mental health (Thakkar et al., 2024).

The implications of these findings highlight the need to include organizational climate in the analysis of mental health, especially in educational and virtual contexts. Organizational climate, defined as the quality of the social and structural environment in which learning takes place, can act as a determining factor in the mental health of students by directly influencing their level of engagement. Thus, a positive organizational climate, whether in physical or virtual environments, fosters engagement, which in turn functions as a key mediator to reduce stress, anxiety, and other challenges related to mental health. Conversely, an unfavorable climate in virtual environments can intensify negative effects on well-being, underscoring the importance of designing inclusive, sustainable, and emotionally safe educational environments. This reinforces the need for institutional policies and practices that prioritize a healthy organizational climate as an integral part of strategies to promote mental health in university students.

Organizational climate is defined as the perception shared by the members of an organization about their work environment, which, in this case, is the perception shared by university students about their academic environment, significantly influencing their performance and well-being. This variable consists of four theoretical dimensions: support climate, goal climate, innovation climate, and rule climate (García-Buades et al., 2015; Mañas-Rodríguez et al., 2020; Mendoza Llanos, 2015).

An interesting study aimed at characterizing the organizational climate of six universities in Colombia found that from the student’s perspective, the organizational climate was grouped into four categories: academic, socio-affective, administrative, and ethical. The results showed that, according to student perceptions, the organizational climate at the university is a set of tangible factors (infrastructure, resources, etc.) and intangible factors (values, interpersonal relationships, situations, etc.) that are experienced on a day-to-day basis (Bermúdez-Aponte et al., 2015). In this sense, organizational climate is an important concept in academic organizations, as it directly influences student experience and satisfaction (Viđak et al., 2024), This is reinforced by another study that has found that organizational climate positively affects student behavior (Wu & Xu, 2022).

The support climate refers to the availability of resources and emotional support from teachers and peers; the innovation climate reflects an openness to new ideas and approaches in the learning process; the goal climate focuses on clarity and commitment to academic objectives; and the rule climate, which encompasses the rules and expectations that regulate academic behavior within the institution. A positive organizational climate favors both the personal and academic development of students, promoting an environment conducive to learning and growth.

Despite the growing relevance of the variables of positive mental health, engagement, and organizational climate in educational settings, research on their interrelationship has been scarce, particularly in the university setting. This study therefore proposes to investigate the interrelationships between these variables through a structural equation model, to provide empirical evidence that will enrich the understanding of their interconnectedness and their impact on the student experience. The research questions, hypotheses, and specific objectives of the study are presented below.

Question: What is the impact of positive mental health on engagement in the educational context?

**H1:**  *Positive mental health has a positive and significant impact on engagement.*

Objective: To determine the impact of positive mental health on engagement in the educational context.

Question: How does the organizational climate influence the positive mental health of participants?

**H2:**  *Organizational climate has a positive impact on the positive mental health of students or teachers*.

Objective: To identify the effect of organizational climate on positive mental health, considering specific components such as innovation, support, goals, and rules.

Question: What is the impact of organizational climate on the level of engagement of participants?

**H3:**  *Organizational climate has a positive and direct impact on engagement, partially mediated by mental health*.

Objective: To examine the direct and indirect relationship between organizational climate and engagement, considering the mediating role of mental health.

## 2. Materials and Methods

### 2.1. Study Design, Variables, and Sample

This quantitative study, with a non-experimental and cross-sectional design, was conducted during one academic semester in 2024. The study had a correlational scope. Three variables were involved in this study: positive mental health, organizational climate, and engagement, and a summary of their main dimensions is shown in Table 1.

Non-probability purposive sampling was applied, and the study sample consisted of 1971 university students, between 16 and 56 years old, with a mean age of 21.09 years, and a standard deviation of 3.2.

The main inclusion criterion consisted of being enrolled in the current academic semester and being willing to voluntarily participate in the study. This decision responds to several methodological and ethical reasons. First, this selection ensures the relevance and timeliness of the data collected, since the students are immersed in the academic and organizational dynamics of the university during that period. This allows us to obtain relevant and contextualized information reflecting current and ongoing university experience. In addition, choosing students enrolled in the current semester guarantees accessibility to the target population and the coherence of the study, since these students share a common academic context, which minimizes heterogeneity in the external conditions that could influence the variables studied. This prevents the results from being influenced by students in different academic situations, such as those who have already completed their studies or those who have not yet started the semester.

The participant gave informed consent. It was voluntary and was self-administered. All participants were previously informed about the nature of the study, its objectives, and how their data would be used. They were assured that their participation was completely voluntary and that they could withdraw from their studies at any time without consequences for their academic status. This transparency respected the participants’ right to make informed and autonomous decisions about their participation.

### 2.2. Data Collection Instruments

This study employed three previously validated scales, ensuring their reliability and validity based on prior research. The instruments used were as follows:

Positive Mental Health Scale (PMH-Scale)

This unidimensional scale, consisting of 9 items, measures positive emotional and psychosocial well-being. The dimensions assessed include happiness, enjoyment, life satisfaction, self-confidence, self-efficacy, holistic well-being, resilience, cheerfulness, and serenity. A 7-point Likert scale was used for this study (1 = strongly disagree; 7 = strongly agree). Example item: “I enjoy my life.” (Lukat et al., 2016; Boufellous et al., 2023).

Utrecht Work Engagement Scale (UWES)—Short Version

The short version of this questionnaire consists of 9 items measuring three dimensions: absorption, dedication, and vigor. A 7-point Likert scale was applied (1 = strongly disagree; 7 = strongly agree). Example item: “I am enthusiastic about my job” —vigor (Schaufeli et al., 2006).

FOCUS Organizational Climate Questionnaire

This questionnaire includes 12 items distributed across four dimensions: support climate, goal climate, innovation climate, and rule climate. A 7-point Likert scale was used (1 = strongly disagree; 7 = strongly agree). Example item: “Suggestions aimed at improving service efficiency and quality are well-received” —innovation (García-Buades et al., 2015; Mañas-Rodríguez et al., 2020; Mendoza Llanos, 2015).

It is important to note that the questionnaires were validated in the language used for the study, ensuring their linguistic and cultural adequacy. The three study instruments—positive mental health (0.910), organizational climate (0.874), and engagement (0.870)—showed good consistency, as well as in all dimensions, except for the rule dimension (0.640), which shows an acceptable level of internal consistency (Table 2).

### 2.3. Fieldwork

The fieldwork was carried out in person in June 2024, using an anonymous, self-administered questionnaire. This modality allowed for greater precision in data collection by reducing interviewer bias and guaranteeing the privacy of the participants. In addition, by integrating the three scales into a single instrument, the process was facilitated for the students, optimizing time and ensuring consistency in the evaluation of the key variables of the study (mental health, academic engagement, and organizational climate). The face-to-face nature also guaranteed adequate control over the administration of the questionnaire, minimizing possible errors.

### 2.4. Data Analysis

The analysis was carried out using the statistical programs AMOS v24, SPSS v26, and R-Project v4.2.2. The data did not contain missing values or outliers in the study. First, the descriptive values of the study’s data were obtained, and then the reliability analysis of the instruments was performed using Cronbach’s Alpha, Theta Coefficient, and Omega statistics, followed by the univariate normality test (Kolmogorov–Smirnov) and multivariate test (Mardia, Royston, Henze-Zirkler and Energy index) of the data that resulted in the non-approximation to a normal univariate and multivariate distribution, respectively, and finally the bivariate correlation test was obtained using Spearman’s correlation coefficient. Secondly, the structural equation model (SEM) was estimated using the asymptotic free distribution estimation method, due to the violation of the multivariate normality assumption in the data in the three study variables. The fit indices used in the estimation of the SEM were the normalized Fit Index (NFI), Goodness-of-Fit Index (GFI), Comparative Fit Index (CFI), Tucker–Lewis Index (TLI), Incremental Fit Index (IFI), Adjusted GFI (AGFI), Relative Fit Index (RFI), Root Mean Square Error of Approximation (RMSEA) and Root Mean Square Error (RMR).

The “acceptable limit” for each indicator was interpreted based on the measures published in an insightful study (Gupta, 2015), detailing the fit criterion (poor fit, ideal fit, perfect fit, good fit, marginal fit, not/less acceptable). These criteria establish standards for evaluating the quality of the model, describing acceptability levels that allow interpretation of the values obtained in each Fit Index.

The values for NFI, GFI, CFI, TLI, IFI, and RFI ranging from 0.80 to 0.90 indicate marginal fit, values between 0.90 and 0.95 suggest good fit, and values ≥0.95 represent perfect fit. Similarly, AGFI values between 0.80 and 0.90 indicate marginal fit, while values ≥0.95 indicate perfect fit. For RMSEA, values between 0.08 and 0.10 indicate marginal fit, values between 0.05 and 0.08 suggest good fit, and values ≤0.05 reflect perfect fit. Lastly, RMR values between 0.05 and 0.08 signify marginal fit, while values ≤0.05 indicate perfect fit (Gupta, 2015).

In more detail, the Gupta (2015) study notes that the absolute fit indices assess how well the model reproduces the observed data:

GFI (Goodness-of-Fit Index): ideal if ≥0.95; good fit between 0.90 and 0.95; marginal fit between 0.80 and 0.90; not acceptable if <0.80.

AGFI (Adjusted Goodness-of-Fit Index): ideal if ≥0.90; good fit between 0.80 and 0.90; not acceptable if <0.80.

The misfit indices measure the degree of error in the model:

RMR (Root Mean Square Residual): ideal if ≤0.05; marginal fit between 0.05 and 0.08; not acceptable if >0.08.

RMSEA (Root Mean Square Error of Approximation): ideal if ≤0.05; marginal fit between 0.05 and 0.08; not acceptable if >0.10.

The incremental fit indices compare the proposed model with a baseline or null model:

NFI (Normed Fit Index), RFI (Relative Fit Index), IFI (Incremental Fit Index), TLI (Tucker–Lewis Index), CFI (Comparative Fit Index): ideal if ≥0.95; a good fit between 0.90 and 0.95; not acceptable if <0.90.

## 3. Results

The average responses of the variables and dimensions range from 4.74 to 5.23 points, with standard deviations ranging from 0.85 to 1.48 points. In addition, all variables and dimensions (indicators) do not approximate the univariate normal distribution (Table 3).

The estimation of the structural equation model was performed using the asymptotic free distribution estimation method because the data did not approximate the multivariate normal distribution (Table 4).

**Figure 1:**
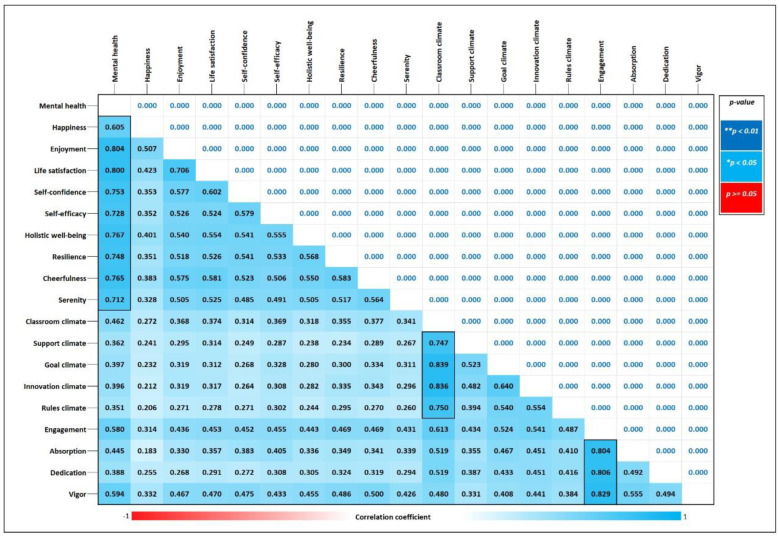
Spearman’s correlation test between variables and dimensions.

Figure 1 shows that the internal correlations between the variables and the dimensions are high, except for the positive mental health variable, with the happiness dimension, which presents an acceptable correlation (0.605). On the other hand, low external correlations are observed between the variables and the dimensions.

### Structural Equation Model (SEM)

For the estimation of the structural equation model, the mental health variable is composed of nine indicators, the organizational climate variable is composed of four dimensions, and the engagement variable is composed of three dimensions, all of which are summarized in the corresponding averages of their items. On the other hand, the data of the variables under study do not present a multivariate normal distribution, since the p-values are less than the theoretical significance value of 0.05. Therefore, the estimation of the structural equation model was carried out using the asymptotic free distribution estimation method, since it is an estimation method that does not require compliance with the multivariate normality assumption.

Table 5 shows that all the indicators of the positive mental health variable are linearly positively related, with a predominance in the life satisfaction dimension (0.768) and a lower weight in the happiness dimension (0.514). Likewise, all the dimensions of the organizational climate variable are linearly positively related, with predominance in the Innovation climate dimension (0.819) and lower weight in the support-climate dimension (0.684). In addition, all the dimensions of the engagement variable are linearly positively related, with a predominance in the vigor dimension (0.814) and a lower weight in the dedication dimension (0.699).

On the other hand, the organizational climate variable is positively related to positive mental health (0.642), positive mental health is positively related to engagement (0.433) and organizational climate is positively related to engagement (0.487).

Finally, covariance between errors means that they have something indirect in common with the corresponding indicators or dimensions that were not measured and that they affect indirectly through their errors.

The Goodness-of-Fit indicators of the structural equation model, assessing the relationship between positive mental health, organizational climate, and engagement, are presented in Table 6, demonstrating that the model adequately fits the observed data according to acceptable criteria.

**Figure 2:**
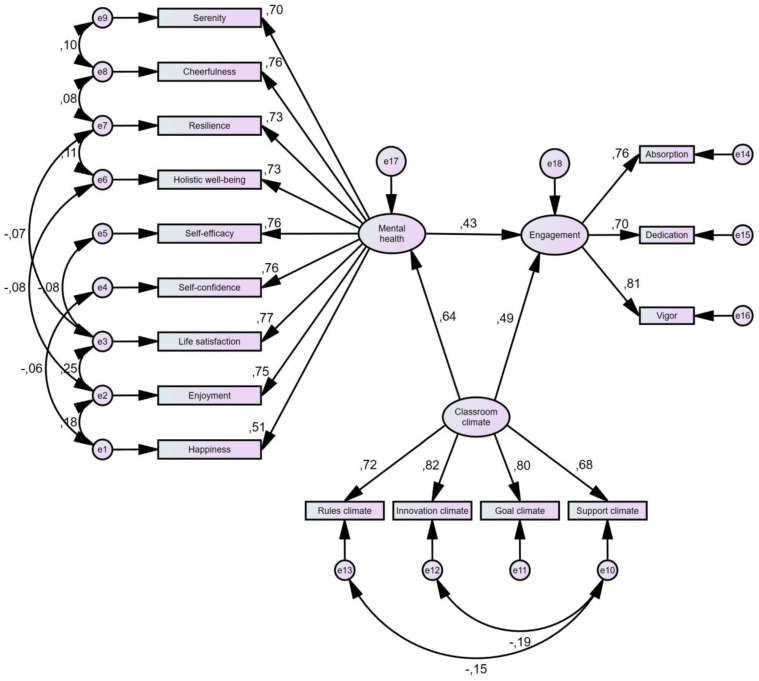
Structural equation modeling of positive mental health, organizational climate, and engagement. Note: standardized coefficients.

According to the fit indicators, it can be said that the estimated structural model is acceptable since it meets four of the Goodness-of-Fit indicators; the model established can be seen below (Figure 2).

## 4. Discussion

Studies that have developed structural equation modeling (SEM) have also found significant interactions between positive mental health and climate in university students (Peoples et al., 2022), determining that organizational climate has a direct influence on learning (Wang & Wang, 2024) and the motivation and commitment of students (Bardach et al., 2020).

Another SEM study showed that game-based learning, while creating a positive and interactive climate, revealed that a higher degree of student participation (engagement) in program activities facilitated educational achievement and, in turn, improved their psychological well-being (Huen et al., 2016).

Meanwhile, another SEM study on university students in Ecuador found that subjective well-being (positive affect, negative affect, and life satisfaction) is latently related to mental health. That is, although subjective well-being influences mental health, this relationship is complex and is expressed through interconnected factors that are not directly observable but are manifested by underlying variables (Mena-Freire et al., 2023). It would be valuable to explore this topic further, as the connections found in our study could contribute to the debate, suggesting that organizational climate and commitment could explain several of these underlying variables linked to the mental health of university students.

However, this is even more complex, as another SEM study of college students in the United States found that family communication had a significant impact on perceived mental health (Shon & Lee, 2024), which placed a special emphasis on other areas such as the family, which would be interesting to include in future studies on holistic well-being in the university context, while another study in the same context warns about the dangers of stigmatizing beliefs on mental health and suicidal ideation (O’Reilly et al., 2024). Therefore, future studies on mental health must be increasingly holistic, addressing different dimensions of the subject (individual, group, institutional, social).

A recent study advocates the importance of promoting a school architecture that contributes to increasing motivation to learn and improve mental health, this study found, using structural equation modeling (SEM), a relationship between school environment, motivation to learn, and mental health, concluding that each element of the school environment has different effects on student learning and life (Abe & Hayashi, 2024). This is closely related to our study that reveals how university organizational climate directly impacts student mental health and engagement.

We have identified and selected another SEM study that also took place in an academic semester with students (the same period as our study), examining the relationship between engagement and burnout, and have found that self-efficacy is a mediating process (Maricuțoiu & Sulea, 2019) which confirms the importance of psychological capital (self-efficacy, resilience, optimism, hope) and its direct implication on commitment. (Sweetman & Luthans, 2010). This is reinforced by the findings of an SEM study which suggests that promoting academic resilience, academic well-being, and student engagement can be effective strategies for improving educational achievement (Sun & Liu, 2023).

One study shows an interesting area associated with the value of loyalty. The results show that this value increases as students’ engagement in their academic process increases, and that, in turn, both perceived beliefs of academic self-efficacy and affective commitment are predictors of loyalty, through academic commitment (Arroyo & Orozco, 2016). In the context of our study, this information highlights the importance of student loyalty as a factor that may be interrelated with organizational climate and mental health. The finding that loyalty increases with student engagement suggests that a positive academic environment and supportive climate may foster greater student dedication and emotional connection to their institution. Furthermore, the fact that perceived academic self-efficacy beliefs and affective commitment act as predictors of loyalty, mediated by academic engagement, reinforces our premise that a favorable organizational climate not only promotes students’ psychological well-being but also enhances their commitment and loyalty to the educational process. This underscores the need to create strategies that strengthen both self-efficacy and affective engagement to enhance academic experience and foster student loyalty, which can have positive repercussions on their mental health and academic performance.

Findings from another study using structural equation modeling reveal the positive impact of students’ emotional intelligence and academic motivation on engagement in learning in the educational setting by adopting teacher support as a mediating variable (Peng et al., 2024). Within the framework of our study, the identification of teacher support as a mediating variable suggests that an educational environment characterized by emotional and academic support from teachers (supportive climate) can enhance students’ emotional intelligence and motivation, which, in turn, translates into greater engagement with their learning process. This aligns with our findings on the influence of organizational climate on students’ mental health and engagement. The implication is clear: to foster greater engagement, and thus better academic performance and emotional well-being, it is crucial to cultivate a supportive classroom climate conducive to emotional intelligence and motivation. Thus, our findings are complementary in emphasizing that teacher support benefits students’ academic engagement and mental health.

Finally, we highlight a relevant study that examines the relationship between perceived peer support, student participation in academic activities, and life satisfaction, using a structural equation modeling approach (Hakimzadeh et al., 2016). This analysis is especially relevant to our research since the three variables studied are aligned with the dimensions that we explored: supportive climate, as a dimension of organizational climate; dedication (which includes students’ protagonist participation), as a component of engagement; and life satisfaction, which represents a dimension of positive mental health. Both studies have identified significant positive correlations between these factors, which reinforces the idea that a supportive environment promotes student engagement and contributes to their overall well-being. This underscores the importance of fostering positive organizational climates that prioritize support and interaction, as these are fundamental for the integral development and mental health of students. In turn, this evidence supports the relevance of our model, highlighting its relevance in understanding the dynamics that influence student well-being.

The relationship between organizational climate and mental health has gained relevance, especially with recent studies highlighting specific factors such as academic burnout and its link with contextual variables. For example, one study highlights how gender influences burnout, which may enrich the analysis of future research by considering how individual characteristics interact with organizational climate (Fiorilli et al., 2022). Also, an interesting study demonstrates how the international theoretical framework on student burnout has changed in recent years to holistically include aspects such as burnout, mental distance, cognitive impairment, and emotional impairment (Romano et al., 2022). This study has important implications for addressing the mental health of students, as it provides a valid and reliable tool to comprehensively identify the main symptoms of school burnout. By including emotional, cognitive, and behavioral components, the BAT-C allows for a more comprehensive assessment, facilitating early and targeted interventions. This can help design tailored support strategies, improve school resources, and promote an educational environment that reduces stress and promotes students’ psychological well-being. Overall, the findings show the influence of the organizational climate on mental health and engagement in Peruvian students, which is in line with global trends observed in countries such as Italy, China, and Finland. However, it should be noted that the impact of the cultural context may vary and that interventions designed to foster a positive climate should consider local adaptations.

### Limitations and Future Projections

Despite the significant contributions of this study, it is important to recognize some limitations. First, the cross-sectional design used prevents us from establishing definitive causal relationships between the variables studied. Although structural equation models allow us to infer connections between organizational climate, mental health, and engagement, we cannot confirm whether changes in one variable directly result in changes in the others over time. Longitudinal studies would be necessary to verify these relationships more robustly.

Furthermore, the study was conducted in a public university in Lima, which limits the generalizability of the results to other populations or educational contexts. Factors such as organizational culture, student characteristics, and institutional policies may vary significantly among universities. In addition, other important contextual variables, such as family support, self-efficacy, and resilience, which previous studies have pointed out as important factors for students’ mental health, were not included.

Given these limitations, future studies should focus on longitudinal analyses that allow us to observe the evolution of the relationships between organizational climate, mental health, and academic engagement over time. This approach would be useful to identify patterns of causality and deepen the dynamics between the variables studied.

**Figure 3:**
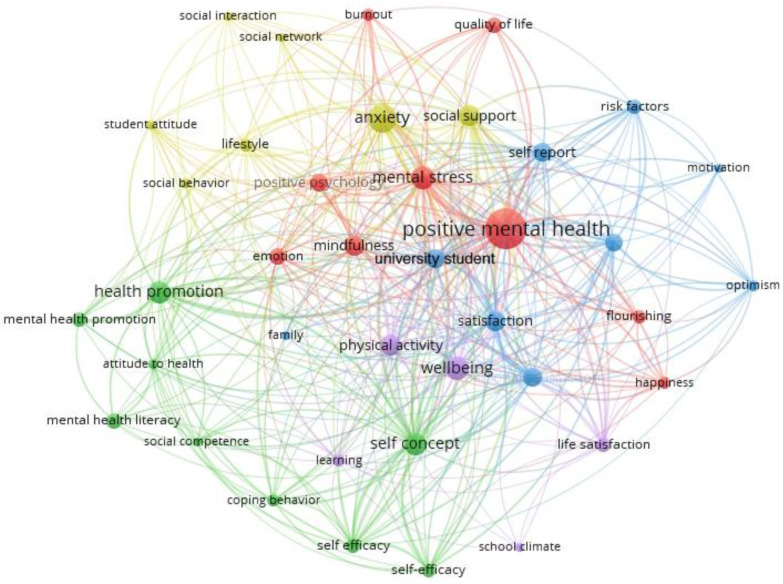
Conceptual network on the positive mental health of college students based on school climate.

Future perspectives of the study suggest the need to consider a comprehensive and multidimensional approach that connects the various factors associated with the positive mental health of students from the school climate as a dynamic base. Next, a cooccurrence network exported from the Scopus database (507 studies) is presented that reveals a dense interrelationship between key concepts such as well-being, stress management, social support, happiness, aspects of psychological capital such as self-efficacy and resilience, as well as behaviors related to mental health promotion, mental health literacy and mindfulness; for future studies, mindfulness can be explored as a structured intervention within universities, assessing its impact on the creation of a healthier organizational climate and its ability to reinforce the connection between emotional well-being, learning and life satisfaction (see Figure 3).

The analysis of this network shows that school climate should be studied not only as an isolated factor but as a central node that interacts with critical dimensions. This opens the door to future research that incorporates more complex methodologies, such as dynamic network analysis, to capture the bidirectional nature of these relationships.

In the future perspectives of the study, it is fundamental to highlight the central role of the concepts identified; for example, in the green cluster, “mental health literacy” emerges as a key component in the promotion of positive mental health. Mental health literacy involves not only knowledge about issues related to psychological well-being but also the ability to recognize, prevent, and effectively manage emotional challenges. In this sense, university environments should foster educational programs that increase students’ competence to understand and act on behalf of their mental health, promoting coping and self-care skills.

In the red cluster, concepts such as “mindfulness”, “flourishing” and “happiness” stand out, which are intrinsically related to positive mental health and subjective well-being. “Mindfulness” is presented as an effective strategy to reduce stress and improve emotional self-regulation, which are crucial aspects in the academic context. On the other hand, “flourishing (understood as a state of optimal functioning in emotional, psychological, and social terms) and “happiness” (associated with satisfaction and the perception of quality of life) should be central objectives in the design of organizational interventions. These interventions could include personal development workshops, emotional well-being programs, and spaces that promote meaningful connections and social support.

In the future perspectives of the study, in addition to the elements highlighted in the green and red clusters, the key concepts in the yellow and blue clusters offer valuable insights that broaden the understanding of the impact of organizational climate on positive mental health and academic engagement.

In the yellow cluster, concepts such as “anxiety”, “mental stress”, and “social support” highlight the importance of addressing the emotional and psychological stress factors that affect students. Anxiety and mental stress are recurrent problems in university contexts, especially in settings where academic demands are high. In this sense, social support is positioned as a critical buffer against these challenges, providing an emotional and practical support network that mitigates the impact of stress on students. Future studies should investigate how university settings can promote a culture of support and collaboration that fosters strong interpersonal relationships, both among students and between students and faculty.

In the blue cluster, concepts such as “well-being”, “satisfaction”, “happiness”, and “flourishing” are linked to positive mental health outcomes. Well-being and satisfaction are associated with a positive perception of quality of life and emotional balance, while “flourishing” reflects an ideal state of functioning in which students not only survive academic demands but thrive in their environment. These concepts suggest that interventions aimed at promoting academic and personal satisfaction, as well as strategies to enhance personal flourishing, are essential for a healthy organizational climate.

In the purple cluster, a direct connection between school climate and essential factors such as learning, life satisfaction, well-being, and positive mental health is highlighted, underscoring its relevance as an area of focus for future studies. School climate, understood as the emotional, social, and organizational environment that characterizes an institution, directly influences the quality of learning by providing a space that promotes motivation, participation, and confidence in students’ abilities.

Likewise, a positive school climate is closely linked to life satisfaction by generating an environment that fosters meaningful interpersonal relationships, individual recognition, and a sense of belonging. This same environment, by prioritizing emotional and psychological well-being, becomes a protective factor for positive mental health, helping to prevent academic stress and promoting personal flourishing.

For future research, we suggest the need to analyze how specific characteristics of school climate (such as teacher support, student participation, and inclusive policies) differentially affect each of these elements. Furthermore, it is suggested to explore the interaction between school climate and individual variables (such as resilience and self-efficacy) to design personalized interventions that enhance learning, well-being, and satisfaction in different educational contexts.

Integrating the findings from these clusters reinforces the importance of a holistic approach that addresses the reduction in stress and anxiety, the enhancement of social support, and the promotion of well-being, satisfaction, and flourishing. Future studies should focus on designing strategies based on these dimensions, evaluating how each interacts within the university ecosystem. This holistic approach will enable the creation of educational environments that not only respond to the psychological and social needs of students but also enhance their academic performance and overall quality of life.

Before concluding, it is important to note that for future studies, it is crucial to analyze how technology and artificial intelligence influence students’ positive mental health, school climate, and academic engagement. Technology, although central to everyday dynamics, can generate burnout and depressive symptoms; however, it also has the potential to promote well-being. Research should focus on designing interventions that strengthen emotional self-regulation, resilience, and social connections, and evaluating how to integrate these tools positively in educational settings (Liu & Ma, 2020; Salmela-Aro et al., 2017; Thakkar et al., 2024).

## 5. Conclusions

The present study highlights the relevance of university organizational climate as a key factor in promoting both positive mental health and academic engagement in university students. The results confirm that a climate of innovation and support significantly influences psychosocial well-being and student engagement, highlighting the dimension of life satisfaction as the main contributor to well-being, and vigor as the most relevant facet of academic engagement. These findings suggest that improving the organizational climate, through strategies that foster innovation and teacher support, could mitigate academic stress, and at the same time optimize academic performance and strengthen the emotional well-being of students, which would be a true educational success.

In addition, the structural analysis reveals a direct connection between organizational climate and mental health, and between the latter and academic engagement. Thus, the study provides empirical evidence of the importance of institutional interventions focused on creating a positive classroom environment that favors holistic well-being, student loyalty, and academic engagement.

According to the structural model, positive mental health is a key factor that directly affects students’ engagement, so implementing strategies that promote mental well-being could improve their dedication, absorption, and vigor in academic activities. The results show that mental health acts as an integrating axis of key variables such as resilience, life satisfaction, self-efficacy, and integral well-being. Together, these factors shape a mental state that enhances students’ ability to fully engage in their academic activities.

Finally, it highlights the need to address the positive mental health of university students from a more holistic and strategic approach, where the organizational climate is reconfigured as a critical factor for human development in universities.

## Figures and Tables

**Table 1:** Variables and dimensions.

Variable	Dimensions
Organizational Climate	Support climate
	Goal climate
	Innovation climate
	Rule climate
Positive Mental Health	Happiness
	Enjoyment
	Life satisfaction
	Self-confidence
	Self-efficacy
	Holistic well-being
	Resilience
	Cheerfulness
	Serenity
Engagement	Absorption
	Dedication
	Vigor

**Table 2:** Measures of instrument reliability.

Variable/Dimension	Number of Items	Cronbach’s Alpha	Coefficient	
			Theta	Omega
Organizational Climate	12	0.874	0.876	0.868
Support climate	3	0.745	0.746	0.855
Goal climate	3	0.731	0.731	0.848
Innovation climate	3	0.753	0.754	0.859
Rule climate	3	0.640	0.642	0.807
Positive Mental Health	9	0.910	0.912	0.927
Engagement	9	0.870	0.871	0.862
Absorption	3	0.805	0.806	0.885
Dedication	3	0.796	0.796	0.880
Vigor	3	0.799	0.799	0.882

**Table 3:** Descriptive characteristics of the study variables.

Variable/Dimension	Media	Standard Deviation	Kolmogorov–Smirnov	
			Statistic	Sig.
**Organizacional Climate**	**4.88**	**0.85**	**0.056**	**0.000**
Support climate	4.96	1.10	0.125	0.000
Goal climate	4.83	1.03	0.090	0.000
Innovation climate	4.85	1.10	0.102	0.000
Rule climate	4.89	0.99	0.090	0.000
**Positive Mental Health**	**5.03**	**1.07**	**0.068**	**0.000**
Happiness	4.74	1.48	0.176	0.000
Enjoyment	5.10	1.44	0.190	0.000
Life satisfaction	5.08	1.43	0.186	0.000
Self-confidence	5.13	1.40	0.193	0.000
Self-efficacy	5.23	1.26	0.209	0.000
Holistic well-being	4.87	1.44	0.189	0.000
Resilience	4.85	1.40	0.190	0.000
Cheerfulness	5.06	1.34	0.191	0.000
Serenity	5.21	1.43	0.208	0.000
**Engagement**	**4.99**	**0.94**	**0.071**	**0.000**
Absorption	5.18	1.08	0.126	0.000
Dedication	4.81	1.15	0.098	0.000
Vigor	4.98	1.17	0.111	0.000

**Table 4:** Multivariate normality test of the data.

Test		Organizational Climate		Engagement		Positive Mental Health	
		Statistic	*p*-Value	Statistic	*p*-Value	Statistic	*p*-Value
Mardia	Asymmetry	2,437,876	<0.001	1945.317	<0.001	1,808,437	<0.001
	Kurtosis	66,871	<0.001	67.941	<0.001	77,683	<0.001
Royston		1,714,159	<0.001	1360.180	<0.001	1,436,055	<0.001
Henze-Zirkler		2258	<0.001	5.206	<0.001	7119	<0.001
Energy		20,542	<0.001	30.924	<0.001	37,515	<0.001

**Table 5:** Structural equation modeling of positive mental health, organizational climate, and engagement.

Relation (←)/Covariability (↔)			Regression Weights		S.E.	C.R.	*p*
			Estimate	Standardized			
Positive mental health	←	Organizational climate	0.637	0.642	0.039	16.290	***
Engagement	←	Positive mental health	0.483	0.433	0.046	10.541	***
Engagement	←	Organizational climate	0.539	0.487	0.042	12.923	***
Happiness	←	Positive mental health	1.000	0.514			
Enjoyment	←	Positive mental health	1.420	0.752	0.061	23.405	***
Life satisfaction	←	Positive mental health	1.423	0.768	0.064	22.226	***
Self-confidence	←	Positive mental health	1.352	0.757	0.062	21.844	***
Self-efficacy	←	Positive mental health	1.256	0.758	0.059	21.428	***
Holistic well-being	←	Positive mental health	1.384	0.729	0.065	21.355	***
Resilience	←	Positive mental health	1.366	0.735	0.066	20.551	***
Cheerfulness	←	Positive mental health	1.349	0.759	0.061	22.129	***
Serenity	←	Positive mental health	1.324	0.698	0.066	20.205	***
Support climate	←	Organizational climate	1.000	0.684			
Goal climate	←	Organizational climate	1.108	0.799	0.041	27.170	***
Innovation Climate	←	Organizational climate	1.193	0.819	0.042	28.357	***
Rules climate	←	Organizational climate	0.952	0.725	0.037	25.469	***
Absorption	←	Engagement	1.000	0.765			
Dedication	←	Engagement	0.940	0.699	0.034	27.695	***
Vigor	←	Engagement	1.121	0.814	0.035	31.715	***
e1	↔	e2	0.202	0.184	0.030	6.831	***
e1	↔	e4	−0.063	−0.061	0.029	−2.162	0.031
e2	↔	e3	0.195	0.249	0.028	6.979	***
e2	↔	e6	−0.072	−0.084	0.025	−2.859	0.004
e3	↔	e5	−0.056	−0.082	0.022	−2.500	0.012
e3	↔	e7	−0.054	−0.068	0.024	−2.227	0.026
e6	↔	e7	0.094	0.109	0.029	3.201	0.001
e7	↔	e8	0.066	0.085	0.028	2.381	0.017
e8	↔	e9	0.086	0.104	0.026	3.290	0.001
e10	↔	e12	−0.089	−0.185	0.018	−4.849	***
e10	↔	e13	−0.080	−0.154	0.017	−4.599	***

Note: e = estimation errors; C.R. = critical region; S.E. = standard deviation. *** Significance at 0.1%.

**Table 6:** Goodness-of-Fit indicators of the structural equation model of positive mental health, organizational climate, and engagement.

Name	Measure of Adjustment	Value	Acceptable Limit
Normalized goodness-of-fit index	NFI	0.817	≥0.90
Goodness-of-fit index	GFI	0.947	≥0.90
Comparative fit index	CFI	0.867	≥0.90
Tucker–Lewis index	TLI	0.822	≥0.90
Incremental goodness-of-fit index	IFI	0.87	≥0.90
Adjusted goodness-of-fit index	AGFI	0.92	≥0.80
Relative goodness-of-fit index	RFI	0.756	≥0.90
Mean square error of approximation	RMSEA	0.032	≤0.05
Square root of the mean square error	RMR	0.094	≤0.08

## Data Availability

The data supporting the conclusions of this article can be provided by the corresponding author upon reasonable request and university ethics approval.
